# Early-Life Exposure to Benzo[a]pyrene Increases Mutant Frequency in Spermatogenic Cells in Adulthood

**DOI:** 10.1371/journal.pone.0087439

**Published:** 2014-01-29

**Authors:** Guogang Xu, C. Alex McMahan, Christi A. Walter

**Affiliations:** 1 Department of Cellular and Structural Biology, the University of Texas Health Science Center at San Antonio, San Antonio, Texas, United States of America; 2 Department of Pathology, the University of Texas Health Science Center at San Antonio, San Antonio, Texas, United States of America; 3 Cancer Therapy and Research Center, the University of Texas Health Science Center at San Antonio, San Antonio, Texas, United States of America; 4 Barshop Institute for Longevity and Aging Sciences, the University of Texas Health Science Center at San Antonio, San Antonio, Texas, United States of America; 5 South Texas Veteran's Health Care System, San Antonio, Texas, United States of America; Montana State University, United States of America

## Abstract

Children are vulnerable to environmental mutagens, and the developing germline could also be affected. However, little is known about whether exposure to environmental mutagens in childhood will result in increased germline mutations in subsequent adult life. In the present study, male transgenic *lacI* mice at different ages (7, 25 and 60 days old) were treated with a known environmental mutagen (benzo[a]pyrene, B[a]P) at different doses (0, 50, 200 or 300 mg/kg body weight). Mutant frequency was then determined in a meiotic cell type (pachytene spermatocyte), a post-meiotic cell type (round spermatid) and epididymal spermatozoa after at least one cycle of spermatogenesis. Our results show that 1) mice treated with B[a]P at 7 or 25 days old, both being pre-adult ages, had significantly increased mutant frequencies in all spermatogenic cell types tested when they were 60 days old; 2) spermatogenic cells from mice treated before puberty were more susceptible to B[a]P-associated mutagenesis compared to adult mice; and 3) unexpectedly, epididymal spermatozoa had the highest mutant frequency among the spermatogenic cell types tested. These data show that pre-adult exposure to B[a]P increases the male germline mutant frequency in young adulthood. The data demonstrate that exposure to environmental genotoxins at different life phases (e.g., pre-adult and adult) can have differential effects on reproductive health.

## Introduction

Benzo[a]pyrene (B[a]P) is a ubiquitous polycyclic aromatic hydrocarbon (PAH) and environmental mutagen. Primary exposure to PAHs results from inhalation of tobacco smoke, wood smoke and ambient air, and consumption of PAHs in foods [1], [2]. Cigarette mainstream smoke contains a variety of PAHs with reported concentrations of B[a]P ranging from approximately 5 to 80 ng/cigarette; sidestream smoke concentrations are significantly higher with sidestream/mainstream concentration ratios for B[a]P ranging from 2.5 to 20 [1]. B[a]P is metabolized to the reactive form (+)-B[a]P-7,8-diol-9,10-epoxide (BPDE), which requires recycling of PAH-diols through the microsomal monooxygenase system [3]. BPDE causes DNA adducts, predominantly at the exocyclic amino groups of guanine and adenine [4], [5], [6]. These BPDE-DNA adducts are associated with the induction of somatic mutations and various cancers [4], [7], [8], [9], [10].

BPDE-DNA adducts are found in spermatozoa of men exposed to cigarette smoke [11], and of adult animals treated with B[a]P [12], [13]. B[a]P-induced male germline mutations were reported using a *lacI* transgenic mutation reporter model [12], [13], in dominant lethal assays [14], and in an expanded simple tandem repeats (ESTR) assay after exposure to tobacco smoke [15] or to PAHs [16], [17], [18]. Therefore, B[a]P is a male germ cell mutagen that has the potential to adversely compromise the health of future generations.

Germline mutations occurring in pre-pubertal males have a substantially greater potential impact on reproductive health because young males have a longer reproductive life. Furthermore, at very young ages there are relatively few spermatogonial stem cells in the testes that will proliferate and sustain spermatogenesis as the male matures sexually. A mutation that occurs in the male germline soon after birth may be expanded as the numbers of spermatogonial stem cells increase. The pre-pubertal stage of development is vulnerable to environmental toxicants [19], [20], [21], [22]. Some exposures to environmental toxicants, e.g., secondhand smoke, in childhood can increase the risk of diseases in later life. Childhood exposure to secondhand smoke is associated with increased lung cancer risk [23], [24], more respiratory symptoms and poorer lung function during adulthood [25]. Approximately 25% of children aged 4 to 11 years and 20% of children aged 12 to 19 years in the United States are exposed to secondhand smoke in the home [26]. It is unknown whether exposure to B[a]P during different life phases (e.g., neonatal, pubertal or adult) impacts adult germline mutagenesis similarly or differently.

In the present study, a *Lac I* transgenic mouse model was used to determine directly if exposure to B[a]P at different life phases has differential effects on mutant frequencies in spermatogenic cells obtained subsequently from adult mice. The *Lac I* transgenic mouse genome carries the bacteriophage λ genome as a transgene, which in turn carries the *Lac I* repressor gene and the α*Lac Z* gene from the prokaryotic *Lac* operon [27]. λ DNA is recovered from mouse genome and used to infect *Escherichia coli* carrying a *Lac Z* (β-galactosidase) gene, but lacking a functional *Lac I* gene. Mutations occurring in the *Lac I* gene can be identified as blue plaques on agarose containing the chromogenic substrate 5-bromo-4-chloro-3-indolyl-β-D-galactopyranoside (X-gal). Each cell in the transgenic mouse, including germ cells carries about 40 copies of *Lac I* gene in a head-to-tail concatemer. This mutagenesis assay is able to identify various mutation classes in different tissues occurring spontaneously [28], [29], [30] or induced by chemicals including BaP [12], [31] and is particularly sensitive to point mutations, such as those induced by BaP. Accordingly, the *Lac I* transgenic mouse model is a powerful system for detecting in vivo mutations in virtually all tissues and cell types at all ages.

Our findings demonstrate that pre-adult exposure to B[a]P can have a significantly greater impact than adult exposure on germline mutagenesis and thereby potentially increase the risk of genetic diseases in offspring.

## Materials and Methods

### Animals

Adult male and female homozygous *Lac I* transgenic mice (C57BL/6) were obtained from Taconic (Hudson, NY) and then mated to generate male homozygous *Lac I* mice. This study was carried out in strict accordance with the recommendations in the Guide for the Care and Use of Laboratory Animals of the National Institutes of Health. The protocol was approved by the Committee on the Ethics of Animal Experiments of the University of Texas Health Science Center at San Antonio (protocol number 02119-34-01-A).

### Benzo[a]pyrene (B[a]P) Treatment

B[a]P (Fluka Chemical Corp., Milwaukee WI) was ground into a fine powder using a mortar and pestle prior to dissolving it in DMSO. Male mice at different ages (7, 25 and 60 days old) were administered various B[a]P doses, i.e., 50, 200 or 300 mg/kg body weight or DMSO solvent alone as a control, as a single intraperitoneal injection. There were 12 groups in total. Each age and dose group consisted of 5 male mice. Mice were humanely euthanized at 60 days old for groups treated at 7 or 25 days old and at 95 days old for groups treated at 60 days old. The interval between the treatment and termination was to ensure that the spermatogenic cells collected for mutation analysis were derived from treated spermatogonial stem cells. The timing was based on the reported length of time (35 days) to complete one spermatogenic cycle [32].

### Preparation of Spermatogenic Cell Types

Pachytene spermatocytes and round spermatids were prepared from 5 male mice using a STA PUT gradient system as described previously [33]. Epididymal spermatozoa were obtained from whole epididymis. Enriched spermatogenic cells were then snap-frozen in liquid nitrogen and stored at −80°C until use for DNA isolation. Liver was collected to serve as a somatic control tissue in the mutagenesis assay.

### DNA Isolation and Mutagenesis Assay

High molecular weight DNA isolation using the RecoverEase™ DNA isolation kit followed the manufacturer's instructions (Agilent Technologies, Santa Clara, CA) as described previously [34]. Lambda phage shuttle vectors harboring the bacterial *lacI* gene were recovered from high molecular weight DNA samples using Agilent Technologies' Transpack *in vitro* packaging extracts. Packaged phage were mixed with *E. coli* SCS-8 cells and added to top agarose containing 5-bromo-4-chloro-3-indoyl-betagalactopyranoside (X-gal) and plated on NZY agar trays. Plaque-forming units (pfus) were counted after incubation overnight at 37°C. Putative blue mutant plaques were visually identified and confirmed by coring and plating again. Mutant frequency was determined by dividing the number of confirmed mutant plaques by the total number of pfus obtained for the sample.

### Statistical Analysis

The numbers of mutants were described by a Poisson regression model with parameter estimates obtained by the method of maximum likelihood [35]. The statistical model included the effects of age, treatment dose, cell type, and all two-factor interactions, and three-factor interactions. The likelihood ration test was used to test for statistically significant differences. Computations were made using SAS PROC GENMOD (Version 9.2, SAS Institute, Cary, NC). P≤0.05 was considered statistically significant.

## Results

### Pre-pubertal Exposure to B[a]P

Pachytene spermatocytes (meiotic cells), round spermatids (post-meiotic cells) and epididymal spermatozoa were collected to assess B[a]P-induced mutant frequencies (MFs) after exposure during the neonatal period. Pachytene spermatocytes and round spermatids displayed significantly increased MFs in the 200 and 300 mg B[a]P/kg body weight treatment groups compared to the control group (i.e., 0 mg B[a]P/kg body weight), p<0.05 (Table 1). Epididymal spermatozoa and liver tissue showed significantly elevated MFs in all B[a]P-treatment groups compared to the control group (Table 1). Notably, MFs in spermatozoa were significantly higher than those in pachytene spermatocytes, round spermatids and liver tissues in the same B[a]P-treatment groups obtained from the same mice, p<0.05. The 300 mg dose of B[a]P further significantly increased MF in spermatozoa compared to the 200 mg B[a]P/kg body weight group, however, not in other germ cells or liver tissue.

**Table 1:** Mutant frequencies (mean ± SE ×10^−5^) in different cell types and liver tissue from mice treated with B[a]P at 7 days of age.

B[a]P		Tissues			
(mg/kg)		Pachy	RS	SP	Liver
0	Pfu	1,036,150	1,106,056	1,119,217	810,810
	Mutants	9	11	19	11
	MF	0.87±0.29	0.99±0.30	1.70±0.39	1.36±0.41
50	Pfu	994,556	1,260,510	1,317,839	858,466
	Mutants	17	15	80	30
	MF	1.71±0.41	1.19±0.31§	6.07±0.68*,§,¶,||	3.49±0.64*
200	Pfu	1,058,352	1,199,365	1,110,481	503,200
	Mutants	35	32	148	35
	MF	3.31±0.56*,§	2.67±0.47*,†,§	13.33±1.10*,†,§,¶,||	6.96±1.18*,†
300	Pfu	1,212,218	1,096,229	1,301,089	856,500
	Mutants	42	41	233	87
	MF	3.46±0.53*,§	3.74±0.58*,†,§	17.98±1.18*,†,‡,§,¶,||	10.16±1.09*,†

Pfu, plaque-forming unit; MF, mutant frequency; Pachy, pachytene spermatocytes; RS, round spermatids; SP, epididymal spermatozoa.

* Significantly (P<0.05) different from 0 mg/kg, same cell type.

† Significantly (P<0.05) different from 50 mg/kg, same cell type.

‡ Significantly (P<0.05) different from 200 mg/kg, same cell type.

§ Significantly (P<0.05) different from liver, same dose.

¶ Significantly (P<0.05) different from pachytene spermatocytes, same dose.

|| Significantly (P<0.05) different from round spermatids, same dose.

### Pubertal Exposure to B[a]P

25 day old mice represent the pubertal period [36]. In contrast to observations in pre-pubertal mice, MF in pachytene spermatocytes was significantly increased only in the 300 mg B[a]P/kg body weight treatment group compared to the control group, p<0.05 (Table 2). MFs for round spermatids were similar across different B[a]P-treatment groups and the control group. However, epididymal spermatozoa and liver tissue showed significantly increased MFs in all B[a]P-treatment groups compared to the control group. MFs were not increased further with increased B[a]P doses for other germ cells or liver.

**Table 2:** Mutant frequencies (mean ± SE ×10^−5^) in different cell types and liver tissue from mice treated with B[a]P at 25 days old.

B[a]P		Tissues			
(mg/kg)		Pachy	RS	SP	Liver
0	Pfu	1,035,568	1,173,356	1,125,084	835,690
	Mutants	9	13	15	15
	MF	0.87±0.29	1.11±0.31	1.33±0.34	1.79±0.46
50	Pfu	1,056,400	958,677	1,066,617	1,616,501
	Mutants	12	14	34	119
	MF	1.14±0.33‡	1.46±0.39‡	3.19±0.55*,‡,§	7.36±0.67*
200	Pfu	1,218,450	915,443	1,269,390	949,651
	Mutants	25	17	42	91
	MF	2.05±0.41‡	1.86±0.45‡	3.31±0.51*,‡	9.58±1.00*
300	Pfu	1,111,772	1,251,366	1,072,640	1,065,230
	Mutants	26	25	37	112
	MF	2.34±0.46*,‡	2.00±0.40‡	3.45±0.57*,‡	10.51±0.99*,†

Pfu, plaque-forming unit; MF, mutant frequency; Pachy, pachytene spermatocytes; RS, round spermatids; SP, epididymal spermatozoa.

* Significantly (P<0.05) different from 0 mg/kg, same cell type.

† Significantly (P<0.05) different from 50 mg/kg, same cell type.

‡ Significantly (P<0.05) different from liver, same dose.

§ Significantly (P<0.05) different from pachytene spermatocytes, same dose.

### Adult Exposure to B[a]P

MFs for pachytene spermatocytes and round spermatids collected from B[a]P-treated adult mice were similar among doses and the control group (Table 3) except for pachytene spermatocytes collected from mice treated with 300 mg B[a]P/kg body weight, in which case MF was significantly elevated. MF in epididymal spermatozoa in the 300 mg B[a]P/kg body weight group was significantly greater than in the 0, 50 and 200 mg B[a]P/kg body weight groups, p<0.05. MFs in liver tissue in the 200 and 300 mg B[a]P/kg body weight groups were significantly elevated compared with the 0 and 50 mg B[a]P/kg body weight groups, p<0.05.

**Table 3:** Mutant frequencies (mean ± SE ×10^−5^) in different cell types and liver tissue from mice treated with B[a]P at 60 days old.

B[a]P		Tissues			
(mg/kg)		Pachy	RS	SP	Liver
0	Pfu	1,282,604	1,075,886	1,058,615	773,650
	Mutants	15	11	18	15
	MF	1.17±0.30	1.02±0.31	1.70±0.40	1.94±0.50
50	Pfu	1,037,256	1,225,971	1,038,088	894,660
	Mutants	8	13	20	24
	MF	0.77±0.27§	1.06±0.29§	1.93±0.43	2.68±0.55
200	Pfu	1,271,560	1,100,110	1,026,400	929,300
	Mutants	15	12	30	59
	MF	1.18±0.30§	1.09±0.29§	2.92±0.53§,¶,||	6.35±0.83*,†
300	Pfu	1,205,650	1,164,790	1,023,638	693,933
	Mutants	24	18	56	53
	MF	1.99±0.41*,§	1.55±0.36§	5.47±0.73*,†,‡,¶,||	7.64±1.05*,†

Pfu, plaque-forming unit; MF, mutant frequency; Pachy, pachytene spermatocytes; RS, round spermatids; SP, epididymal spermatozoa.

* Significantly (P<0.05) different from 0 mg/kg, same cell type.

† Significantly (P<0.05) different from 50 mg/kg, same cell type.

‡ Significantly (P<0.05) different from 200 mg/kg, same cell type.

§ Significantly (P<0.05) different from liver, same dose.

¶ Significantly (P<0.05) different from pachytene spermatocytes, same dose.

|| Significantly (P<0.05) different from round spermatids, same dose.

Comparisons among spermatogenic cells showed that MFs in spermatozoa were significantly greater in the 200 and 300 mg B[a]P/kg body weight groups than in pachytene spermatocytes and round spermatids within the same dose group, p<0.05. MFs in somatic tissue (i.e., liver) in all B[a]P treated groups were significantly greater than in spermatogenic cell types, except for spermatozoa in the 50 mg B[a]P/kg body weight group, which was similar to MF in liver.

### Age and B[a]P Mutagenesis

**Figure 1:**
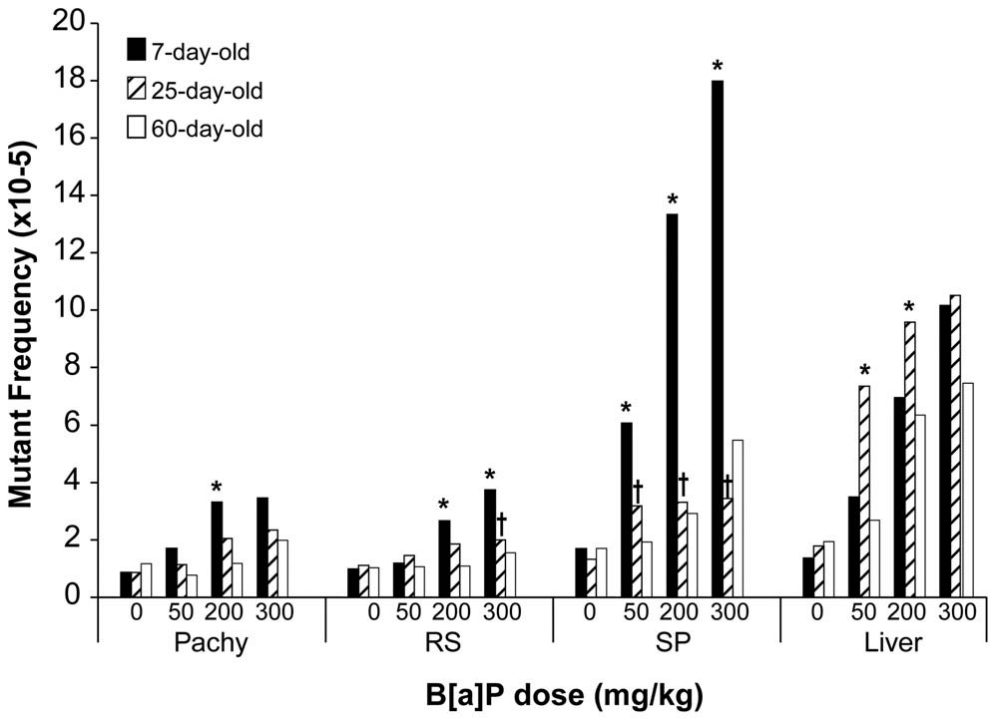
Comparisons of mutant frequencies (MFs) in spermatogenic cells and liver among different age groups. The data are presented as means (×10^−5^) only. Standard errors are listed in Tables 1–3 and are not presented in the Figure for clarity. * Significantly (P<0.05) greater than in 60 day old mice, same cell type and same dose. † Significantly (P<0.05) greater than in 25 day old mice, same cell type and same dose. Pachy, pachytene spermatocytes; RS, round spermatids; SP, epididymal spermatozoa.

Pre-pubertal exposure to B[a]P caused elevated MFs in all three tested spermatogenic cell types compared to pubertal and adult exposures (Fig. 1). Significantly increased MFs were observed in epididymal spermatozoa from 7 day old mice in all B[a]P-treated groups compared to 25 and 60 day old treatment groups, p<0.05. Pachytene spermatocytes and round spermatids showed significantly greater MFs in 7 day old mice treated with 200 mg/kg of B[a]P than comparable cells in similarly treated 60 day old mice, p<0.05. In addition, round spermatids from mice treated pre-pubertally displayed significantly increased MFs compared to pubertal and adult mice at the highest B[a]P dose group, p<0.05. In contrast, MFs for all three spermatogenic cell types were similar between 25 day old mice and 60 day old mice.

Liver MFs were significantly elevated for mice treated with B[a]P at 25 days old compared to adult mice in the 50 and 200 mg B[a]P/kg body weight groups, p<0.05 (Fig. 1). Liver MFs were similar to germ cell types among the different age groups at all other B[a]P doses. Thus, pre-pubertal exposure to B[a]P did not significantly increase the prevalence of mutations in adult liver compared to pubertal or adult exposure to B[a]P.

## Discussion

Children are susceptible to environmental mutagens and carcinogens [19], [20], [21], and a variety of adult diseases have been associated with early life environmental exposures [23], [24], [25], [37], [38] [39]. The present study using mice as a model demonstrates that early life exposure to B[a]P, particularly pre-pubertal exposure, causes significantly increased MFs in the male germline in later life compared to adulthood exposure. Thus, there are developmental time periods that render the male germline more susceptible to B[a]P-induced mutagenesis. The most susceptible age tested was 7 days old, a time when spermatogonia are proliferating in the immature mouse testis.

The mechanism that renders pre-pubertal mice more susceptible to B[a]P-induced germline mutagenesis is not clear. Tight junctions develop between adjacent Sertoli cells to form a blood-testis barrier [40], [41], but this barrier has not yet formed in 7 day old mice. After the barrier forms, spermatogonial stem cells reside in the basal compartment of seminiferous tubules and are on the unprotected side of the tight junctions [42]. Thus, pre-pubertal spermatogonia are presumably no more accessible to B[a]P than are adult spermatogonial stem cells. Consequently, it seems reasonable that other mechanisms are involved in the greater mutagenic response of pre-pubertal spermatogonia to B[a]P.

A potential mechanism is a differential metabolic capacity in pre-pubertal and adult spermatogonial stem cells. B[a]P and other PAHs cause DNA damage after enzymatic biotransformation by components of the cytochrome P450 system such as CYP1A1, CYP1A2, CYP1B1, into reactive intermediates (reviewed in [43]). Hepatic CYP1A1 and CYP1A2 are expressed at the highest levels in pre-pubertal male rats compared to adult rats [44]. CYP1A1 is expressed in human testes [45] and B[a]P significantly induced the expression of CYP1A1 in multiple tissues including the gonads in goldfish (*Carassius auratus*) [46]. It is not known if CYP1A1 is expressed at a greater level in spermatogonial stem cells of pre-pubertal animals, but greater expression would result theoretically in greater bioactivation of B[a]P. Consequently, more DNA damage could be realized in pre-pubertal spermatogonial cells.

A second potential mechanism involves cellular responses to DNA damage, e.g., DNA repair. B[a]P-induced DNA damage, e.g., BPDE-DNA adducts, are repaired largely by the nucleotide excision repair (NER) pathway [47], [48]. We [49] and others [50] have reported that male germ cells have limited NER activity compared to somatic cells. In our study [49], type A spermatogonial cells from 8 day old mice were able to remove only 60% of UV-induced photoproducts compared to their complete removal by keratinocytes within a 12 h period. Whether young adult spermatogonial stem cells have a more efficient NER activity than pre-pubertal spermatogonial stem cells warrants further investigation.

A third potential mechanism involves the phagocytic function of Sertoli cells. During spermatogenesis, more than 50% of differentiating spermatogenic cells die through apoptosis [51], [52], and are eventually eliminated through phagocytosis by Sertoli cells [53], [54], [55]. The phagocytic capability of Sertoli cells requires contact between unknown specialized structures on the outer surfaces of the spermatocytes [56]. However, spermatocytes in male mice do not appear until 10 days of age [33]. Therefore, Sertoli cells in 7 day old pre-pubertal mice treated with B[a]P were not fully activated by spermatocytes in terms of their phagocytotic activity, and consequently germ cells with B[a]P-induced DNA damage could not be removed as efficiently by Sertoli cells compared with adult mice. This could potentially result in persistence of cells with a higher mutant frequency.

A fourth potential mechanism involves cell division rates of spermatogonial stem cells. During embryogenesis the prospermatogonia proliferate to sustain the maturing testes. The burst of proliferation, much of it occurring in the first week after birth [57] is a highly susceptible time for mutations to be created because DNA is being replicated and can serve as a source of mutations. Other possible mechanisms, such as those already discussed could potentially exacerbate the replication risk for mutations.

Human males have a much longer pre-pubertal period compared to mice (∼12 years vs. ∼3 weeks) [36]. These 12 years account for about 17% of a lifetime for a 70-year-old human male versus ∼8.3% of a lifetime for a 3 year old male mouse. Type A spermatogonia are the most abundant germ cell type in the testis from birth to puberty in humans [58]. Therefore, human type A spermatogonial cells have a relatively longer timeframe during which the developmentally immature cells can be exposed to environmental agents. Five and six days after the World Trade Center (WTC) disaster, 12.1∼23 µg B[a]P/g dust and smoke was detected in downtown New York City from three undisturbed, protected locations to the east of the WTC site [59]. It took more than 6 months for the airborne concentrations of seven PAHs (including B[a]P) to drop to the background levels at Ground Zero [60]. Children born in areas surrounding the WTC around the time of the disaster are now at pubertal age, and children that were at or near puberty are now in adulthood. The effect of this accidental exposure to relatively high B[a]P levels on their reproductive health is unknown. In the present study, a single dose of B[a]P in pre-pubertal mice resulted in a subsequently increased MF in spermatozoa at adulthood. The ramifications of environmental exposures to developmentally immature gonads may include significant reproductive consequences later in life.

The spermatogenic cell types used to measure B[a]P-induced MFs in the present study descended from B[a]P-exposed spermatogonial stem cells [32]. Therefore, it is expected that the increased MFs reflect a greater mutant frequency in the treated stem cell populations. During spermatogenesis, stem cells self-renew and differentiate to give rise to spermatozoa, which carry a complete set of paternal genetic and epigenetic information for development of the next generation. The increased MF in spermatogonial stem cells due to B[a]P exposure during early life would theoretically result in spermatozoa with a greater MF throughout the male reproductive life because these are the stem cells that will sustain spermatogenesis. This, in turn, would result in an increased risk of adverse reproductive outcomes in the exposed generation and genetic diseases in their offspring.

Spermatogenic cells from mice treated with B[a]P as adults displayed a lower MF compared to liver. Our results are consistent with these previous studies in which lower MFs in spermatogenic cells were also observed compared to liver when treated with B[a]P in adults [12], [13]. However, in the present study, MFs for spermatozoa were significantly greater than those for liver obtained from the same mice treated with B[a]P before puberty. This finding demonstrates the vulnerability of pre-pubertal spermatogonial stem cells to B[a]P exposure. The results demonstrate that there is also a period during postnatal development when the liver is more susceptible to B[a]P-induced mutagenesis. Mice treated with 50 and 200 mg/kg B[a]P at 25 days old, during the pubertal period, displayed a significantly increased mutant frequency in liver compared with mice treated at 7 or 60 days old.

Mice treated with ionizing radiations (IR) during pre-puberty, i.e., 6 or 8 days old, showed significantly increased MFs in type A spermatogonial cells at 8 days of age or in spermatocytes at 18 days, compared to untreated mice [61]. However, the increased MFs returned to background levels at later stages of spermatogenesis (including epididymal spermatozoa) obtained from 30 or 60 day old mice after IR treatment [61]. A reduction of spontaneous MFs in spermatogenic cells during the first wave of murine spermatogenesis was reported previously [34]. The mechanism(s) for reduction of IR-induced MFs and spontaneous MFs during spermatogenesis is not clear. However, the MF remained high in BaP treated animals as indicated in the present study. The same *lacI* transgene was used as a reporter gene for mutagenesis in previous studies [34], [49] and in the present study, thus it is not a difference in sensitivity of the reporter gene used. Another possibility might reside with the type of mutation that is generated. Spontaneous mutations in the germline are largely C to T transitions in the *lacI* [30], [62], [63], whereas there is an increased prevalence of G to T transversions in *lacI* genes in IR treated *E. coli* cells [64] and B[a]P-treated mice [12], [65]. It is also possible that the type of DNA damage is important in determining whether spermatogenic cells are able to elicit a reduction in mutant frequency. B[a]P creates bulky lesions in the DNA and these lesions are repaired largely through the nucleotide excision repair pathway. This pathway is less effective in spermatogenic cells than in somatic cells [49], [50]. Thus, it is possible that the signaling pathway required to mediate reduced mutant frequency is not generated if the lesions are repaired largely through nucleotide excision repair.

The present study has clearly demonstrated that pre-pubertal exposure to B[a]P significantly increased mutant frequency in male germ cells during subsequent adulthood in mice. The route of exposure to BaP in humans is typically ingestion or inhalation, not by intraperitoneal injection. Studies in mice have shown that ingestion and intraperitoneal injection both lead to germline effects [12], [66], [67], [68], [69]. Therefore, differences in effects between these two routes of exposure seem minimal. Human exposures in events such as the World Trade Center can reach 12.1–23 µg B[a]P/g dust [59]. Using conversion factors to assess the doses used in mice in the present study it is determined that the mice were challenged with the equivalent of 4–24 mg/kg body weight (or 4–24 ppm) in humans [70]. These similarities between mouse and humans suggest the possibility that human childhood exposure to certain germ cell mutagens could have a significant impact on adult male reproductive health and warrants further investigation.
